# Role of paracoccin on *Paracoccidioides brasiliensis* virulence and susceptibility to antifungal drugs in the *Galleria mellonella* larvae model

**DOI:** 10.1080/21505594.2022.2150455

**Published:** 2023-01-04

**Authors:** Nayla de Souza Pitangui, Fabrício Freitas Fernandes, Thiago Aparecido da Silva, Relber Aguiar Gonçales, Maria Cristina Roque-Barreira

**Affiliations:** Department of Cell and Molecular Biology and Pathogenic Bioagents, Ribeirão Preto Medical School, University of São Paulo, Ribeirão Preto, São Paulo, Brazil

**Keywords:** *P. brasiliensis*, Paracoccin, susceptibility, resistance, fungal cell wall

## Abstract

Paracoccin (PCN), a *Paracoccidioides brasiliensis* glycoprotein, has been reported to play roles in fungal biology and paracoccidioidomycosis pathogenesis. Lectin and chitinase domains account for the PCN’s dual roles as an immunomodulatory agent and virulence factor. Soluble PCN injected in *P. brasiliensis* infected mice, by interacting with TLRs’ N-glycans, drives the host immune response toward a protective Th1 axis. Otherwise, mice infection with yeasts overexpressing PCN (ov-PCN) revealed that PCN acts as a fungal virulence factor, thanks to its chitinase activity on the cell wall, resulting in resistance to phagocytes’ fungicidal activity and development of severe paracoccidioidomycosis. Because antifungal drug administration follows the disease diagnosis, we studied the PCN effect on yeast resistance or susceptibility to antifungal agents. Using a paracoccidioidomycosis model developed in *Galleria mellonella* larvae, we confirmed the observation, in the murine host, that ov-PCN yeasts display maximum virulence compared to wild-type (wt-PCN) or PCN-silenced (kd-PCN) yeasts. PCN overexpression accounted for the highest susceptibility of *P. brasiliensis* to antifungal and reduced relative mRNA expression of genes encoding proteins related to cell wall remodeling. The lowest virulence, detected in infection with kd-PCN yeasts, correlated with the lowest susceptibility to antifungals and impact on genes for cell wall remodeling. So, we defined that the grade of endogenous PCN production influences the *P. brasiliensis* virulence and susceptibility to antifungal drugs, as well as the expression of genes related to cell wall remodeling. We postulate that this variable gene expression is mechanistically associated with *P. brasiliensis* virulence changes.

## Introduction

Paracoccin (PCN) is a virulence factor contributing to the establishment of *Paracoccidioides brasiliensis* infection. In the last decade, PCN was identified as a bifunctional protein, whose lectin domain binds to N-acetylglucosamine and chitin [1,2], whereas the enzymatic domain exerts N-acetylglucosaminidase (NAGase) activity. Localized on the surface of *P. brasiliensis* yeast cells, mainly in budding regions, PCN plays a prominent role in fungal growth. Its enzymatic activity is associated with remodeling of the fungal cell wall owing to its ability to hydrolyze multimeric structures (chitin) into monosaccharides (GlcNAc). When subcutaneously injected in *P. brasiliensis* infected mice, native or recombinant PCN modulates, through its lectin activity, the host immune response toward the Th1 (T helper 1) axis, protective against the infection. Nonetheless, mice infection with *P. brasiliensis* yeasts that were transformed to overexpress PCN (ov-PCN) revealed that PCN, through its chitinase activity, acts as a fungal virulence factor. These properties depend, at least partially, on the local offer of substrate for PCN. The subcutaneous tissue, where PCN is injected, glycoproteins abound on the surface of numerous resident macrophages, making possible interactions between the PCN lectin domain and glycans N-linked to toll-like receptors (TLR), which signal for cytokines production, cause macrophages’ M1 polarization, and induce a protective Th1-immune response. Otherwise, infection with ov-PCN yeasts favors that secreted PCN interaction, thorough its enzymatic domain, with chitin of the cell wall. Its intense hydrolysis favors the yeasts resistance to phagocytes’ fungicidal activity, causing severe paracoccidioidomycosis [3–6].

Recent studies have explored the impact of silencing and overexpressing the PCN gene (PADG_03347) on *Paracoccidioides* spp. yeasts. Both transformed yeasts underwent significant changes in PCN expression compared to the wild type (WT, wild type). On the one hand, PCN silencing results in the following *in vitro* effects: (a) reduced frequency of individual yeast cells, (b) blocking of the yeast-mycelium transition, and (c) augmented susceptibility to macrophages’ fungicidal activity. Mice infection with these PCN-silenced yeasts courses with a reduction of (a) pulmonary fungal burden, (b) tissue damage, and (c) mortality rate [7]. On the other hand, PCN overexpression leads yeasts to the following *in vitro* modifications: (a) a shorter and more efficient yeast-mycelium transition and (b) augmented yeast resistance to macrophages’ fungicidal activity. *In vivo* studies showed that mice infected with ov-PCN transformants present (a) increased pulmonary fungal burden, (b) more severe tissue injury, and (c) augmented mortality rate of the infected mice [8]. Therefore, there is sufficient evidence that PCN plays a relevant role in the pathogenesis of paracoccidioidomycosis, acting as a virulence factor for *P. brasiliensis* yeasts. PCN impact on the virulence of yeasts of the *Paracoccidioides* complex is well-established. However, the effects of PCN on yeast resistance to antifungal pharmacological agents used to treat human paracoccidioidomycosis remain unknown. To clarify this point is the main objective of the present study.

## Methods

### *P. brasiliensis* strains

*P. brasiliensis* wild-type strain (wt-PCN), PCN-overregulated strain (ov-PCN), PCN-downregulated strain (kd-PCN), and empty vector (EV) were maintained in solid or liquid brain heart infusion (BHI) media (Kasvi, São José dos Pinhais, Brazil) supplemented with 1% glucose at 37 °C on a mechanical shaker (200 rpm).

### Construction of PCN-overexpressing and PCN-silenced *P. brasiliensis* yeasts

To obtain PCN-overexpressing and PCN-silenced *P. brasiliensis* strains, we used the antisense RNA (AsRNA) strategy and *Agrobacterium tumefaciens*-mediated transformation (ATMT), as described previously by Fernandes et al [7]. and Gonçales et al [8]. Briefly, to obtain *P. brasiliensis* strains with silenced PCN, two regions (AS1 and AS2) of the second exon of PCN were amplified and individually inserted into the pCR35-RHO2 vector. The pCR35-RHO2 vectors carrying AS1 or AS2 were used for amplification of the AsRNA cassettes and individually inserted into the transfer DNA (T-DNA) of the parental binary vector pUR5750. These vectors were used to transform *A. tumefaciens* LBA1100 cells. The transformants were selected in LB medium containing 100 mg/ml kanamycin and subsequently used for the transformation of *P. brasiliensis* by co-cultivation with yeasts at a ratio of 1:10 in sterile Hybond N filters (GE Healthcare Life Science, Pittsburgh, PA) placed in IM solid medium (induction medium) at 28 °C for 3 days. After co-culture, the membranes were transferred to a liquid BHI medium containing cefotaxime (200 mg/ml). Cells in suspension were incubated for 48 h at 200 rpm and 37 °C before being transferred to selective BHI medium containing hygromycin B (100 mg/ml) and cefotaxime (100 mg/ml) at 37 °C for 15 days. To obtain PCN-overexpressing strains, we used the same methodology; however, the complete gene was inserted into the vector in the sense. The silencing and overexpression of the clones were verified using qRT-PCR. All the mutants were mitotically stable.

### Antifungal drugs

Amphotericin B, itraconazole, and micafungin were obtained from the manufacturer (Sigma-Aldrich, MO, USA) and prepared according to document M27-S4, proposed by the Clinical and Laboratory Standards Institute in 2012 with adaptations for *P. brasiliensis.*

### Cloning, expression in *Pichia pastoris*, and purification of recombinant paracoccin

Recombinant PCN was obtained as described by Freitas et al. [6] Briefly, the PCN ORF, cloned into the pUC57 vector by Alegre et al [3], was amplified with the forward primer 5′-CTCGAGATGGCGTTTGAAACCAGATTG-3′ and reverse primer 5′-GCGGCCGCCCAGCTGCTGGTGCTAAAGC-3′. The purified PCR product was cloned into the pGEM-T vector (Promega, Fitchburg, WI, USA) and subsequently into the pGAPzαA vector (Invitrogen, Carlsbad, CA, USA). The pGAPzαA-PCN vector was used to transform *Pichia pastoris* GS115, as described by Maleki et al. [9] Transformed strains obtained in YPD-selective medium containing Zeocin (100 mg/mL) were confirmed using PCR. The selected clone was cultivated in 300 mL of liquid YPD at 30 °C and 220 rpm for 72 h for expression and secretion of recombinant PCN. The culture supernatant was collected, dialyzed against phosphate buffered saline (PBS, pH 7.2), and concentrated 10 times using centrifugal filtration devices with a 10,000-molecular weight cut-off (Millipore, Darmstadt, Germany). For PCN purification, the culture supernatant was applied to a chitin column as described by Dos Reis Almeida et al [2]. The product obtained from chromatography was analyzed using SDS-PAGE and subjected to quality control of biological activity by evaluating its lectin and enzymatic activities.

### Minimum inhibitory concentration (MIC)

Susceptibility tests of *P. brasiliensis* (wt-PCN, ov-PCN, kd-PCN, and EV) to amphotericin B, itraconazole, and micafungin were performed according to De Paula e Silva et al [10], with adaptations. Inoculi were prepared in Roswell Park Memorial Institute (RPMI-1640) medium (Sigma-Aldrich) with l-glutamine, without sodium bicarbonate, supplemented with 2% glucose, and buffered to a pH of 7.0 using 0.165 M morpholine propane sulfonic acid (MOPS; Sigma-Aldrich), to achieve a final concentration in microdilution plates of 0.5 × 10^5^ colony-forming units (CFU)/ml. For quality control, *Candida parapsilosis* ATCC 22,019 was tested at a concentration of 2.5 × 10^3^ CFU/ml. Work solutions of amphotericin B, itraconazole, and micafungin were tested at concentrations ranging from 0.001955 to 8 μg.ml^−1^, 0.00001525 to 8 μg.ml^−1^, and 0.0156 to 128 μg.ml^−1^, respectively, after adding the inoculum. The plates were incubated at 37 °C with agitation at 150 rpm for 72 h. The readings were obtained using Alamar Blue® (Sigma-Aldrich). The lowest antifungal agent concentration that substantially inhibited the growth of the organism was visually determined at the point where there was no change in the original blue color of the reagent. Three independent experiments were conducted.

### Minimum fungicide concentration (MFC)

Qualitative analysis of fungal viability was performed by transferring a portion of the wells to a plate with BHI agar (Kasvi) and incubating at 37 °C for 7–10 days. The minimum fungicide concentration was determined as the lowest concentration of the compound that did not allow the growth of any fungal colony on solid medium after the incubation period. Visual reading was performed [11]. Three independent experiments were conducted.

### Analysis of the impact of antifungals on yeast morphology

The antifungal action on PCN-overexpressing and PCN-silenced *P. brasiliensis* yeast cells was evaluated through microscopy using an inverted microscope equipped for observation in a bright field (Leica DMI 6000 B, Wetzlar, Germany). Yeasts that were not subjected to the action of drugs were used as controls.

### Cell wall stress susceptibility

An ideal way to determine susceptibility to stressors is to inoculate a series of yeast concentrations in the form of spots on plates containing the stressor [12]. For the stress susceptibility analysis, 72-h old *P. brasiliensis* yeast cells (wt-PCN, ov-PCN, kd-PCN, and EV) were adjusted to concentrations of 10^7^, 10^6^, and 10^5^ cells/mL and spotted onto plates with BHI agar (Kasvi) supplemented with different stressor agents at different concentrations as follows: calcofluor white (CFW, 20 μg.ml^−1^ and 40 μg.ml^−1^), Congo red (CR, 25 μg.ml^−1^ and 50 μg.ml^−1^), sodium chloride (NaCl, 100 mM and 200 mM), hydrogen peroxide (H_2_O_2_, 5 mM and 7.5 mM), and sodium dodecyl sulfate (SDS, 0.005% and 0.010%). The control plates did not contain any stressor. The plates were incubated for 7–10 days at 37 °C before being photographed. Additionally, the susceptibility of *P. brasiliensis* strains to cellular stress-inducing agents was tested *in vitro* by MIC determination according to document M27-S4 [13]. Work solutions of CFW, CR, NaCl, H_2_O_2_ and SDS were tested at concentrations ranged that from 0.078 to 40 μg.ml^−1^, 0.097 to 50 μg.ml^−1^, 0.781 to 400 mM, 0.097 to 50 mM, and 0.0000195 to 0.010%, respectively, after adding the inoculum. The impact of stressors on the yeast cell wall was evaluated using an inverted microscope equipped for bright-field observation (Leica DMI 6000 B; Wetzlar, Germany). Yeasts that were not exposed to stressors were used as controls.

### Insects

*Galleria mellonela* larvae were kept in a glass container at 25 °C in the dark and fed a specific manipulated feed. For the assays, larvae without color changes and with adequate weight (150–200 mg) were selected and kept without food in petri dishes at 37 °C in the dark for 24 h prior to infection.

### Galleria mellonella *infection*

Larval infection was performed as described by Singulani et al [14]. Prior to the injection, an area of the pro-leg was sanitized with 70% alcohol. The larvae were then inoculated using a Hamilton syringe (Hamilton Co., Reno, NV, USA) in the last left leg. Each group of larvae was inoculated with 10 µL of wild-type (wt-PCN), transformant (ov-PCN or kd-PCN), or empty vector (EV) strains of *P. brasiliensis* (5 × 10^6^ cells/larva). In all assays, a group of uninfected larvae and a group of larvae inoculated with PBS were used as controls. Eight larvae were used for each condition, and each experiment was repeated thrice.

### Survival assay

Infected larvae were incubated in petri dishes at 37 °C and evaluated for 12 days due to a lack of physical movement (motility) and melanization.

### Fungal burden

At 48 h post-infection, the larvae from each group were superficially disinfected with 70% ethanol. Then, through puncture of the abdomen of the larvae, haemolymph samples were collected and diluted in ice-cold PBS with 20 mg/L ampicillin (1:10). Then, 100 µL aliquots of the haemocyte suspension were plated on BHI agar supplemented with 1% glucose and 4% foetal bovine serum (FBS) containing 100 µg/ml ampicillin. The plates were incubated at 37 °C for 10 days. At the end of this period, the CFU were counted.

### Haemocyte density

At 48 h post-infection with wild-type and transformant strains of *P. brasiliensis*, haemolymph samples were collected by puncturing the larval abdomen and diluted in ice-cold PBS (1:20). Then, 10 µL aliquots of the haemocyte suspension were counted using a Neubauer haemocytometer.

### In vivo *resistance assays*

At 1 h after infection with *P. brasiliensis*, larvae were inoculated with amphotericin B (0.5 mg/kg) in the last right pro-leg. Amphotericin B stock solutions were prepared in dimethyl sulfoxide (DMSO) (Labsynth, Diadema, SP, Brazil) and diluted in PBS to a DMSO concentration of 5%, as described previously [14]. A group of uninfected larvae was treated with an antifungal agent alone to assess toxicity. The larvae were incubated at 37 °C for 7 days to prevent physical movement. Additionally, within 48 h of treatment with amphotericin B, haemolymph samples were collected through puncture of the larvae’s abdomen, and haemocyte density was established through microscopy using a Neubauer haemocytometer.

### Histopathological evaluation

At 48 h post-infection and after treatment with amphotericin B (48 h post treatment), the larvae of each group were fixed by immersion in 4% buffered formalin for 24 h. Then, the larvae were preserved in 70% ethanol and longitudinal incisions in the dorsal part were performed with the aid of a scalpel. The samples were dehydrated with increasing concentrations of ethanol, washed with xylene, embedded in paraffin, sectioned serially at a thickness of 5 µm, and stained with periodic acid-Schiff (PAS) (Sigma-Aldrich). Images were acquired using an Olympus microscope comprising the Virtual Slide VS120 system (Olympus, Tokyo, Japan) on a 20× objective and analyzed using the FIJI software (ImageJ; NIH, Bethesda, MD, USA).

### Crude extract preparation, RNA Isolation, cDNA Synthesis, and qPCR Assays

Cultures of wild and transformant *P. brasiliensis* strains were maintained weekly by subculturing on BHI agar (Duchefa, Netherlands) supplemented with 1% glucose and 4% FBS (Life Technologies, Carlsbad, CA, USA) or yeast extract-peptone-dextrose (YPD) agar as described by Pitangui et al. [15] Yeast colonies were then transferred to YPD broth, incubated at 37 °C under agitation (150 rpm) for 72 h, and collected by centrifugation for 10 min (2300 × g, 4 °C). Yeast cells were washed with PBS and total RNA was isolated using TRIzol (Life Technologies) according to the manufacturer’s instructions. The quality and concentration of the RNA samples were determined by using electrophoresis in a 1.2% (w/v) agarose gel with observation of the image in a gel documentation system (Chemidoc MP Imager, Bio-Rad Laboratories, Richmond, CA, USA) and spectrophotometric analysis using NanoDrop® (Thermo Fisher Scientific, Wilmington, DE, USA). RNA samples were treated with DNAse I (Fermentas, Waltham, MA, USA) to remove genomic DNA, and cDNA was synthesized using a cDNA synthesis kit (iScript cDNA Synthesis Kit, Bio-Rad) according to the manufacturer’s instructions. PCR was performed using EVA Green (Bio-Rad) on a CFX96 Real-Time Detection System (Bio-Rad) in a reaction volume of 15 µL under the following conditions: 95 °C for 30 s, followed by 40 cycles of 95 °C for 5 s and 60 °C for 5 s. Gene expression was quantified using the ΔΔCt method in relation to endogenous controls of α-tubulin and L34. The specific gene primers used for the quantitative RT-PCR of fungal cell wall synthesis and degradation markers were *PbFKS1, PbCHS3, PbCSR1, PbNAG1, PbBGN1, PbBGN2*, and *PbAGN*. The primer sequences used in the RT-PCR assays are listed in Table 1. This analysis was conducted as previously described [16].

**Table 1.** Primers used in this study and their target genes.

Approved Gene Symbol	Protein/enzyme	Oligonucleotide Primer Sequence (5’- 3’)
*PbFKS1*	1,3-β-D-glucan synthase	F: TCTGCGGATTTCATTTTGGG R: GTAGATTGGTGGGCGGATTTG
*PbCHS3*	Chitin synthase 3	F: CGCTATGGTTAAGGATCCCGAGA R: GCATCCAGGCAAGCAAGTAACA
*PbCSR1*	Chitin synthase regulator	F: GAAACGACGCCATCATCCAG R: CTCATACGCAGCGCCAAGAC
*PbNAG1*	N-acetyl-β-D-glucosaminidase	F: TTCTGGATAAGTTGATGGCGG R: AAGGTTTTAGGACGTCTCTGC
*PbBGN1*	β-1,3-glucanase	F: GAGAAACTGCTACTGTCCACC R: TGGATGGGATTGGACTTTG
*PbBGN2*	β-1,3-glucanase	F: CCACAGTCCCATTCACATCTC R: GTTGGAAGACTCAGAGGACATG
*PbAGN*	α-1,3-glucanase	F: CAGCAAACACTAAACCCAACG R: CCCTGAACCCACATGTACTAAG
*A-Tub*	α-tubulin	F: CGGCTAATGGAAAATACATGGC R: GTCTTGGCCTTGAGAGATGCAA
*L34*	Ribosomal protein L34	F: CGGCAAACCTCAGATACCTTC R: GGAGACCTGGGAGTATTCACG

F, primer forward.

R, primer reverse.

### Statistical analysis

Data were analyzed using GraphPad Prism v7.00 (GraphPad Software, Inc., La Jolla, CA, USA). Results are expressed as mean ± standard deviation (SD), with observed differences being considered significant at *p* < 0.05 (*). All data are representative of three independent experiments. For the analysis of quantitative RT-PCR data, a One-Way ANOVA test for analysis of variance (ANOVA) test was performed, with Bonferroni’s multiple comparison test.

## Results

### PCN overexpression correlates with high susceptibility of *P. brasiliensis* to antifungal drugs

To investigate whether PCN could influence the susceptibility of *P. brasiliensis* yeasts to antifungal drugs, we evaluated amphotericin B, itraconazole, and micafungin at the lowest concentrations required to inhibit the *in vitro* growth of yeasts differing in PCN expression (wt-PCN, ov-PCN, and kd-PCN). To ascertain the MIC parameter, we cultured yeasts from each strain at equal concentrations (0.5 × 10^5^ CFU/mL) in liquid medium. Test drugs were added to the cultures at several concentrations. After 72 h, the yeast cells were stained with Alamar Blue, which allowed the visualization of the lowest concentration of each drug required to inhibit yeast growth. As shown in Table 2, for each assayed drug, equal or similar MIC values were found for wt-PCN and kd-PCN. These values were at least 2- to 4-fold higher than the MIC achieved by ov-PCN yeasts, indicating that PCN overexpression correlates with a yeast *in vitro* profile of high susceptibility to all assayed antifungal drugs. Yeasts from the wt-PCN and kd-PCN strains displayed low vulnerability to drugs, as shown by the high MIC values determined for all tested drugs.

**Table 2.** Minimum inhibitory concentration (MIC) and minimum fungicidal concentration (MFC) of antifungal drugs against *P. brasiliensis* yeasts from several strains.

	AmB		ITZ		MFG	
	MIC (μg.ml^−1^)	MFC (μg.ml^−1^)	MIC (μg.ml^−1^)	MFC (μg.ml^−1^)	MIC (μg.ml^−1^)	MFC (μg.ml^−1^)
**Strains**						
wt-PCN	0.125	0.125	0.000488	0.000488	>128	>128
ov-PCN	0.0313	0.0313	0.000122	0.000122	64	64
kd-PCN	0.125	0.25	0.000488	0.000976	>128	>128
EV	0.125	0.125	0.000488	0.000488	>128	>128

AmB: amphotericin B; ITZ: itraconazole; MFG: micafungin; MIC: minimum inhibitory concentration; MFC: minimum fungicidal concentration.

wt-PCN: *P. brasiliensis* wild-type strain; ov-PCN: PCN-overregulated strain; kd-PCN: PCN-downregulated strain and; EV: empty vector.

The second parameter used to analyze the susceptibility of yeasts to the tested drugs was the determination of the minimum fungicidal concentration (MFC), which discriminates whether the drug effect verified by MIC has a fungistatic component in addition to a fungicidal mechanism. MFC determination was performed by transferring a part of the well content of the microplate prepared for MIC determination to a plate containing BHI agar. After one week of culturing, the MFC was visually determined as the lowest concentration of the drug that completely prevented the growth of any fungal colony on the plate. The MFC values for each antifungal drug determined for the yeasts of a certain strain were compared with the respective MIC values. The antifungal agents amphotericin B, itraconazole, and micafungin provided MFC equivalent to the MIC values for wt-PCN and ov-PCN yeasts. For the kd-PCN yeasts, the MFC values for amphotericin B and itraconazole were 2-fold higher than the respective MIC values. Precise determination of MIC and MFC for micafungin was hampered by the fact that the highest drug concentration assayed (128 µg.mL^−1^) was not sufficient to completely inhibit the growth of wt-PCN and kd-PCN yeasts. Because 64 µg/mL micafungin was sufficient to block the growth of ov-PCN yeasts, we could determine the MIC and MFC for this strain, for which the values were coincident, and verified that they were at least 2-fold lower than the values of MIC and MFC (>128 µg.mL^−1^) attributed to the other yeast strains.

Our results suggest that a fungistatic component of the tested drugs, amphotericin B and itraconazole, contributed to their apparently better performance when the MIC parameter was determined. We suppose that this contribution is small, because only a slight difference between the MIC and MFC values was detected for amphotericin B and itraconazole.

### PCN overexpression enhances the *P. brasiliensis* cell integrity damage and altered general pattern of yeast organization caused by micafungin

**Figure 1.**
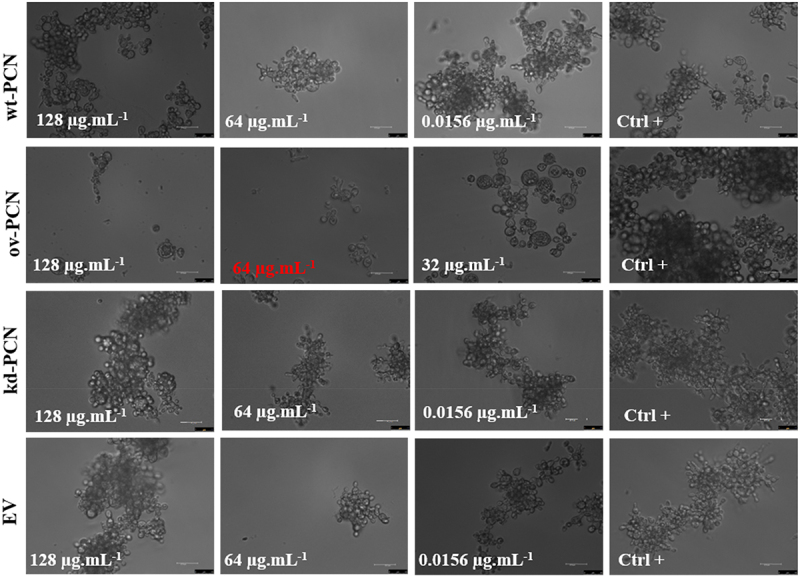
Micafungin differently influences the growth pattern of yeasts from various *P. brasiliensis* strains. Concentration highlighted in red represents the Minimum Inhibitory Concentration (MIC). wt-PCN: *P. brasiliensis* wild-type strain; ov-PCN: PCN-overregulated strain; kd-PCN: PCN-downregulated strain; and EV: empty vector. Ctrl+: Growth control, yeast free from drug effects. 63X objective. Bars, 50 µm.

We performed yeast microscopic analyses to evaluate whether antifungal agents affect the growth pattern of ov-PCN and kd-PCN yeasts compared to that of wt-PCN yeasts. Treatment with amphotericin B or itraconazole did not result in substantial differences in the transformed yeasts compared with the wt-PCN yeasts (data not shown). The yeasts treated with micafungin, in turn, showed distinct growth patterns on ov-PCN compared to wt-PCN and kd-PCN yeasts (Figure 1). Following treatment with 64 µg.mL^−1^ and 128 µg.mL^−1^ micafungin, wt-PCN and kd-PCN yeasts grew by forming aggregates and did not exhibit structural damage to the cell integrity after incubation with micafungin. Otherwise, ov-PCN yeasts grew individually or in smaller aggregates, with reduced number of yeasts, and markedly altered the structural integrity of its cell after contact with the antifungal agent for 72 h (Figure S1).

### PCN overexpression enhances *P. brasiliensis* susceptibility to stress at the cell wall

We evaluated the susceptibility of *P. brasiliensis* yeast (10^5^, 10^6^, and 10^7^ cells/mL) to agents that induce cellular stress (oxidative, osmotic, and structural stress at the membrane or cell wall level). The following stress agents were employed: calcofluor (a chitin ligand), Congo red (a β-1,3-glucan ligand), NaCl (induces osmotic stress, alters ionic homoeostasis), H_2_O_2_ (enhances cellular oxidative processes), and SDS (solubilizes cell membrane lipids). Susceptibility was qualitatively estimated by spotting different yeast concentrations onto plates with a solid medium supplemented with the stressor (at two different concentrations). Yeast growth was estimated by visual examination of the fungal colony size on solid culture medium.

**Figure 2.**
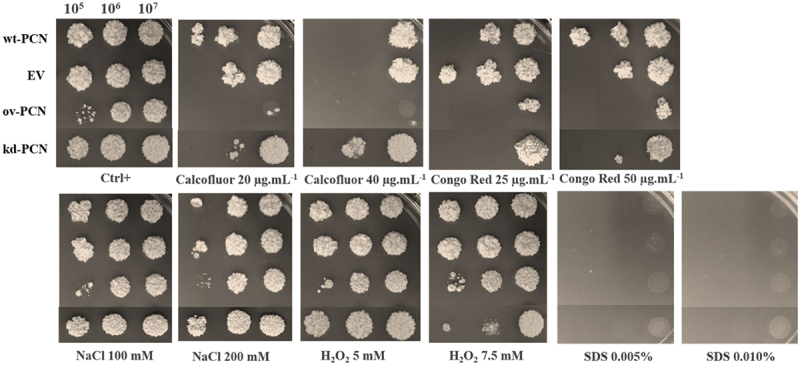
Susceptibility of yeasts from various *P. brasiliensis* strains to cellular stress-inducing agents. The following *P. brasiliensis* strains were assayed: wt-PCN yeasts; ov-PCN yeasts; kd-PCN yeasts; and EV: empty vector. Ctrl+: control of yeasts grown on BHI agar containing 100 µg/ml ampicillin. The plates were incubated at 37 °C for 7–10 days for colony formation. The colonies were visually examined.

Treatment with 100 mM NaCl did not affect the susceptibility of ov-PCN, kd-PCN, and wt-PCN yeasts because the obtained growth profile for each strain was similar to that of the respective yeast positive controls (not subjected to cellular stress) (Figure 2). When 200 mM NaCl was used, there was an augmented susceptibility of yeast 10^5^ concentrated on all strains, except for the kd-yeasts, for which the susceptibility was similar to that observed in the absence of stress. This indicates that when PCN is silenced, yeasts become more resistant to osmotic stress.

By assaying an agent that induces oxidative stress (H_2_O_2_), we verified that when used at 5.0 mM, it did not modify yeast growth, which was visualized in the absence of a stressor (Ctrl+), for each fungal strain. Nonetheless, kd-PCN yeasts (10^5^ and 10^6^ cells/mL) were more susceptible to the effect of 7.5 mM H_2_O_2_.

SDS, the agent that affected the integrity of the fungal cell membrane at both tested concentrations prevented the growth of yeast from all strains.

Yeasts from all strains showed high susceptibility to agents that induced stress at the cell wall level (calcofluor and Congo red). Notably, 40 µg/mL calcofluor was the most potent stressor for the assayed yeasts. The ov-PCN yeasts were more susceptible to the action of calcofluor (20 μg.ml^−1^ and 40 μg.ml−1) and Congo red (25 μg.ml^−1^ and 50 μg.ml^−1^) than the wild-type yeasts. The kd-PCN transformant exhibited particularly increased resistance to the chitin ligand (calcofluor 20 μg.ml^−1^ and 40 μg.ml^−1^) in relation to the profile exhibited by the wild-type strain (wt-PCN) and the PCN-overexpressing strains (ov-PCN). Comparing the strain susceptibility to both stressors of the cell wall, it is noteworthy that, compared to wt-yeasts, ov-PCN and kd-PCN yeasts exhibited minimal and maximal resistance, respectively, to the assayed stressors.

The antifungal activity of the agents inducing cellular stress – calcofluor, Congo Red, NaCl, H_2_O_2_, and SDS – was quantitatively evaluated against yeasts of *P. brasiliensis* strains by determining the MIC, that is, the minimal concentration of a particular agent required to inhibit yeast growth (Table 3). We confirmed that ov-PCN yeasts are more susceptible to cellular stress-inducing agents that act at the cell wall level (calcofluor and Congo red), relative to the susceptibility profile displayed by the wt-PCN yeasts. The MIC value of ov-PCN yeasts toward calcofluor was 5 μg/mL, while the MIC value for the wild-type strain was 10 µg.mL^−1^. In relation to Congo red, the susceptibility of ov-PCN yeasts was even higher than that of the wt-PCN strain, with MIC values <0.097 µg.mL^−1^ for ov-PCN and 0.390 µg.mL^−1^ for wt-PCN. Considering other cellular stress-inducing agents (NaCl, H_2_O_2_ and SDS), it is noted that wt- and ov-PCN yeasts exhibit a similar susceptibility profile.

**Table 3.** Minimum inhibitory concentration (MIC) of cellular stress-inducing agents to inhibit growth of *P. brasiliensis* yeasts from several strains.

	Calcofluor	Congo Red	NaCl	H_2_O_2_	SDS
	MIC (μg.ml^−1^)	MIC (μg.ml^−1^)	MIC (mM)	MIC (mM)	MIC (%)
**Strains**					
wt-PCN	10	0.390	200	0.390	0.00125
ov-PCN	5	<0.097	200	0.390	0.00125
kd-PCN	20	1.562	400	3.125	0.00125
EV	10	0.781	200	0.781	0.0025

NaCl: sodium chloride; H_2_O_2_: hydrogen peroxide; SDS: sodium dodecyl sulphate; MIC: minimum inhibitory concentration.

wt-PCN: *P. brasiliensis* wild-type strain; ov-PCN: PCN-overregulated strain; kd-PCN: PCN-downregulated strain and; EV: empty vector.

Conversely, kd-PCN yeasts were more resistant to cellular stress-inducing agents that act on the cell wall (calcofluor and Congo Red), osmotic stress (NaCl), and stress oxidizing agents (H_2_O_2_) (Table 3). This is reflected in the higher MIC values for kd-PCN yeasts than those exhibited by the wt-PCN and ov-PCN strains. For calcofluor, the MIC of kd-PCN transformants was 20 µg.mL^−1^, in contrast with the values of 10 µg.mL^−1^ and 5 µg.mL^−1^ for wt-PCN and ov-PCN yeasts, respectively. The susceptibility profile to the β-1,3-glucan ligand (Congo red) is represented by MIC values of 0.390 µg.mL^−1^, <0.097 µg.mL^−1^, and 1.562 µg.mL^−1^ for wt-PCN, ov-PCN, and kd-PCN yeasts, respectively. These results suggest that PCN silencing has an impact on the composition of cell wall polysaccharides, chitin, and β-1,3-glucan, and is associated with a dose-dependent increase in yeast resistance to cell wall stressors (calcofluor and Congo Red).

### PCN-overexpression increases the *P. brasiliensis* virulence as verified*in vivo* in a *G. mellonellamodel of fungal infection*

**Figure 3.**
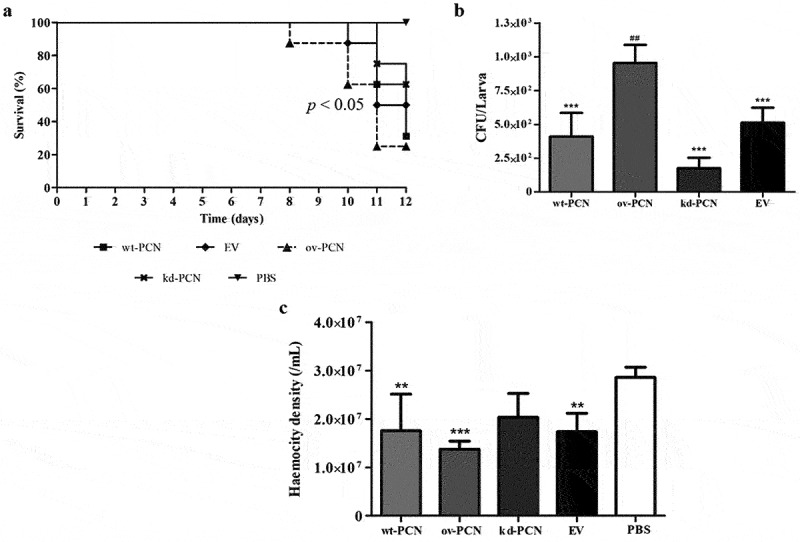
PCN effect on the virulence of *P. brasiliensis* as verified in *G. mellonella* experimental fungal infection. Each *G. mellonella* larva was infected with 5 x 10^6^ yeasts of wt-PCN, ov-PCN, kd-PCN, or EV (empty vector) (*n* = 24). Larvae inoculated with PBS were used as negative controls. (a) Survival curves of infected *G. mellonella* larvae with *P. brasiliensis* yeasts from different strains; *p* values represent the log-rank test in wild-type strain *vs*. transformed yeasts. (b) CFU recovering 48 h after infection of *G. mellonella* larvae with *P. brasiliensis* yeasts from different strains. *** *p* < 0.0001 *vs*. ov-PCN-infected group; **##** *p* < 0.001 *vs*. kd-PCN infected group. (c) Hemocyte density, determined by counting cells in a Neubauer hemocytometer of the hemolymph samples, collected 48 hours after infection with *P. brasiliensis* yeasts from different strains; asterisks indicate statistical significance (*** *p* < 0.0001) relative to the PBS group.

This study included an *in vivo* investigation of the effect of PCN expression on the virulence of *P. brasiliensis* yeasts using a model developed in the invertebrate *G. mellonella*. Larvae infected with different strains of *P. brasiliensis* (wt-PCN, ov-PCN, kd-PCN, and EV) at 5 × 10^6^ yeasts/larva had their survival examined on day 12 after infection. Larval survival was 37.5% among those infected with wt-PCN, 25% with ov-PCN, and 62.5% with kd-PCN (Figure 3(a)).

The fungal burden of the *P. brasiliensis* infected larvae evaluated 12 days after infection, through haemolymph CFU analysis, showed a substantially higher CFU recovery in ov-PCN-infected larvae than in those infected with wt-PCN, kd-PCN, and EV strains. Larvae infected with the three aforementioned strains exhibited similar fungal loads (Figure 3(b)).

The third parameter analyzed in the *G. mellonella* model was the haemocyte density, counted using a Neubauer haemocytometer 48 h after infection with *P. brasiliensis* strains. Microscopic examination showed a 2-fold reduction in the haemocyte density of larvae infected with wt-PCN, ov-PCN, and EV strains compared to the negative control (PBS). kd-PCN-infected larvae had a haemocyte count similar to that of the negative control group (PBS), as shown in Figure 3(c).

**Figure 4.**
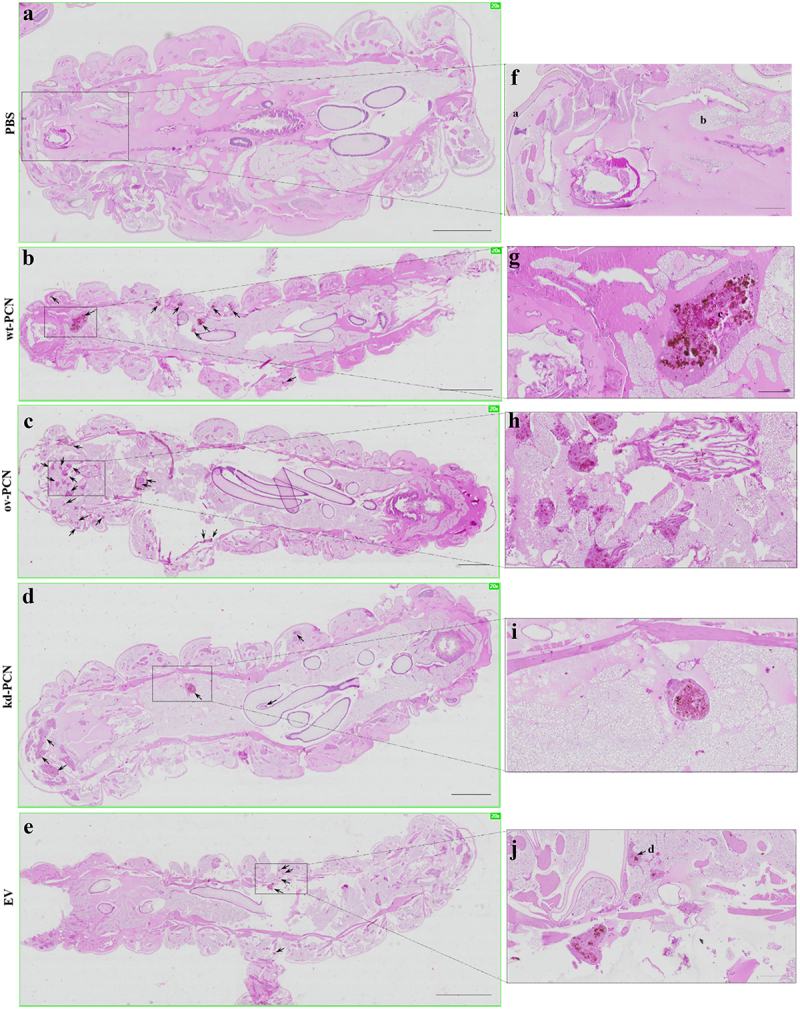
PCN effect on the virulence of *P. brasiliensis* according to histological findings in infected *G. mellonella* larvae. Forty-eight hours after being infected with yeasts from *P. brasiliensis* strains, wt-PCN (b), ov-PCN (c), kd-PCN (d), and EV (e), *G. mellonella* larvae sections were PAS-stained and microscopically examined. Control corresponds to uninfected larvae inoculated with phosphate-buffered saline (PBS) instead of yeasts (a). 20X amplification (a-e). Enlarged images (f-j). Bars indicate 1 mm at A, C, and D; 2 mm in B and E; and 200 µm of F-J. Black arrows indicate *P. brasiliensis* yeast aggregates; a, cuticle; b, adipose bodies; c, granuloma-like structure; d, fungal cells.

We investigated whether PCN-silencing and PCN-overexpression influenced the anatomopathological aspects of experimental infection by *P. brasiliensis* in *G. mellonella*. Larvae infected with yeast from one of the studied *P. brasiliensis* strains (wt-PCN, EV, kd-PCN, and ov-PCN) were euthanized 48 h post-infection. Through microscopic examination of body larvae sections, staining, we verified that in all larvae, independent of the infective *P. brasiliensis* strain, fungal materials were predominantly localized in the periphery of the larvae, close to the cuticle, and the peripheral adipose bodies. We also observed that infection with all *P. brasiliensis* strains showed a similar melanization pattern at the sites of infection. Notably, intracellularly localized fungal material was detected as cell aggregates of variable sizes, indicating mammal granuloma formation (Figure 4). We found a considerable number of these granuloma-like structures in larvae infected with ov-PCN yeasts (Figures 4(c–h)), resulting in wide tissue dissemination, with granuloma-like structures occupying a large area of the larval surface. This was evidenced by comparison with larvae infected with wt-PCN (Figures 4(b–g)), EV (Figures 4(e–j)), and kd-PCN yeasts (Figures 4(d–i)), in which there were fewer granuloma-like structures occupying a lesser larval surface.

### PCN-overexpression decreases the susceptibility to amphotericin B therapy of *G. mellonella* larvae infected with *P. brasiliensis* strains

We assessed the susceptibility profile of *G. mellonella* infected with yeasts from *P. brasiliensis* several strains to the antifungal therapy with amphotericin B, the drug of choice for the treatment of severe cases of human paracoccidioidomycosis.

**Figure 5.**
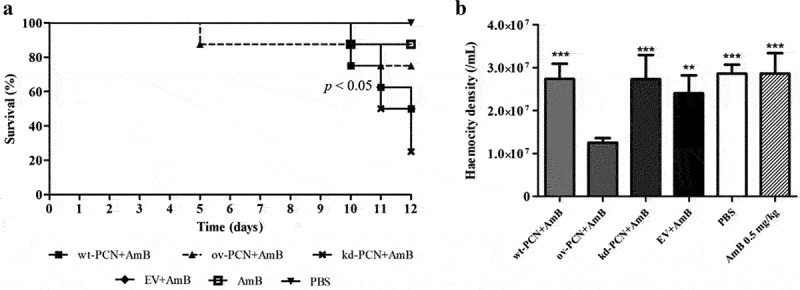
PCN effect on the *P. brasiliensis* susceptibility to amphotericin B (AmB) as verified in *G. mellonella* experimental fungal infection. (a) Survival curves of *G. mellonella* larvae infected with 5 x 10^6^ yeasts from *P. brasiliensis* different strains and treated at 1 h post-infection with amphotericin B 0.5 mg/kg; *p* values represent the log-rank test in wild-type strain treated with AmB *vs*. transformed yeasts treated with AmB. (b) Hemocyte density in hemolymph samples collected 48 h after *P. brasiliensis* infection; asterisks indicate statistical significance (*** *p* < 0.0001) *vs*. ov-PCN+amphotericin B. Larvae inoculated with PBS or amphotericin B were used as controls.

Figure 5(a) shows the survival curves of larvae infected with different strains of *P. brasiliensis* (wt-PCN, ov-PCN, kd-PCN, and EV) and treated with amphotericin B (0.5 mg/kg) administered 1 h after infection. On day 12 after infection, a survival rate of 50% was observed for larvae infected with wt-PCN and EV, 75% for ov-PCN, and 25% for kd-PCN yeasts.

Using the same experimental protocol, we verified that 48 h after infection, haemocyte densities were 2-fold reduced in larvae infected with ov-PCN yeasts. The infection of larvae with wt-PCN, EV, and kd-PCN presented haemocyte density similar to that exhibited by the controls (PBS and amphotericin B 0.5 mg/kg) (Figure 5(b)).

**Figure 6.**
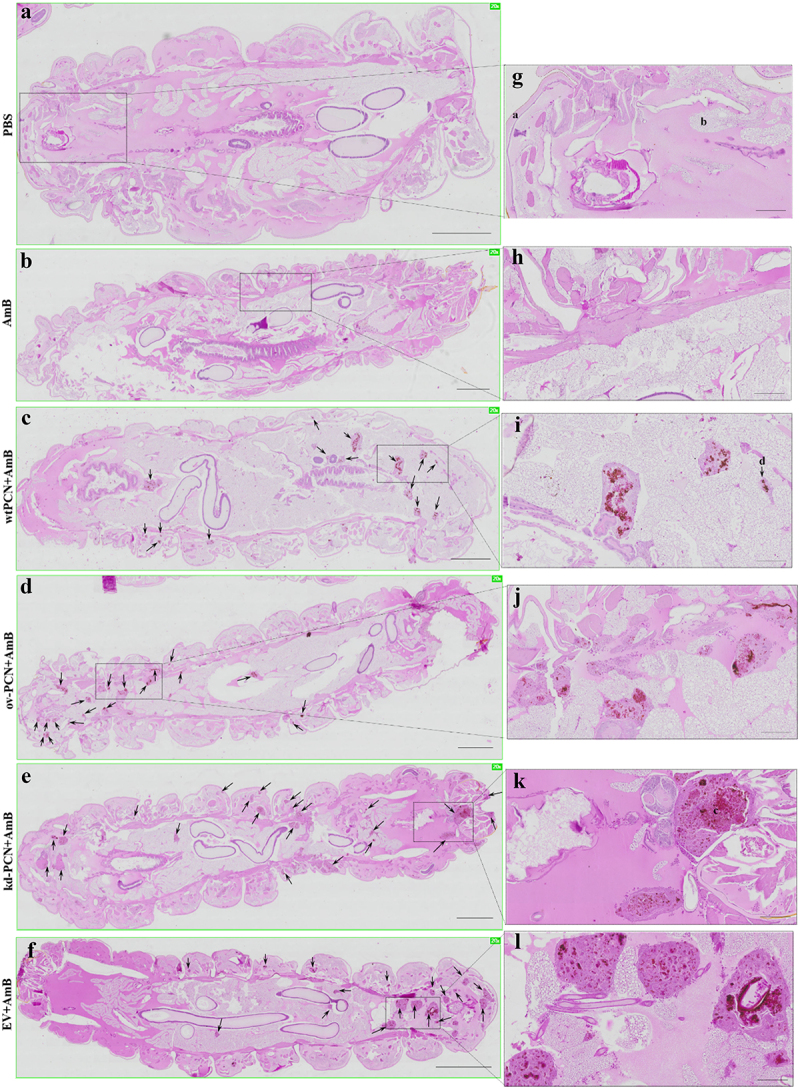
PCN effects on the susceptibility of *P. brasiliensis* yeasts to amphotericin B (AmB), according to histological findings in infected *G. mellonella* larvae. *G. mellonella* larvae were infected with yeasts from different *P. brasiliensis* strains and treated, 1 hour after, with amphotericin B, 0.5 mg/kg. At 48 h post-infection, larvae were euthanized, and body sections were PAS-stained. Images were captured from larvae infected with wt-PCN yeasts (c); ov-PCN yeasts (d); kd-PCN yeasts (e); and EV yeasts (f). PBS: uninfected larvae, inoculated with phosphate-buffered saline (PBS) instead of yeasts (a). Amphotericin B: uninfected larvae inoculated with amphotericin B (0.5 mg/kg) (b). 20X amplification (a-f). Enlarged views of the images (g-l). Bars indicate 1 mm on A-E, 2 mm at F, and 200 µm at G-L. Black arrows indicate *P. brasiliensis* yeast aggregates; a, cuticle; b, adipose bodies; c, granuloma-like structure; d, fungal cells.

We investigated whether *G. mellonella* larvae infected with kd-PCN or ov-PCN *P. brasiliensis* yeasts and treated with amphotericin B presented histological evidence of modified susceptibility to antifungal therapy. Larvae infected with yeasts from one of the studied *P. brasiliensis* strains (wt-PCN, EV, kd-PCN, and ov-PCN) were treated with amphotericin B (0.5 mg/kg) 1 h post-infection and euthanized 48 h post-infection. Figure 6 shows the histological views of *G. mellonella* larvae infected with *P. brasiliensis* and treated with amphotericin B.

Larvae infected with kd-PCN yeasts and treated with amphotericin B exhibited a greater number of fungal aggregates and a larger extent of tissue injury (Figures 6(e–k)) than larvae infected with wt-yeasts and treated with amphotericin B (Figures 6(c–I)). An additional comparison with the histological images of larvae infected with kd-PCN yeasts and not treated with amphotericin B (Figures 6(d–I)) showed that antifungal drug administration was not associated with a reduction in host tissue injury. These histological images reveal that PCN-silencing augments yeast resistance to therapy with amphotericin B.

In turn, larvae infected with ov-PCN yeasts and treated with amphotericin B exhibited focal aggregates of fungal material and granuloma-like structures occupying restricted larval areas (Figures 6(d–j)), compared to the larvae infected with wt-PCN yeasts and treated with amphotericin B (Figures 6(c–I)). An additional comparison between larvae infected with ov-PCN yeasts and those not treated with amphotericin B (Figures 6(c,h)) shows that antifungal drug administration diminishes tissue injury. Put together, these results indicate that PCN overexpression increases yeast susceptibility to amphotericin B therapy.

### PCN-overexpression and PCN-knockdown differently impact the relative mRNA expression of markers related to *P. brasiliensis* yeast cell wall remodelling

**Figure 7.**
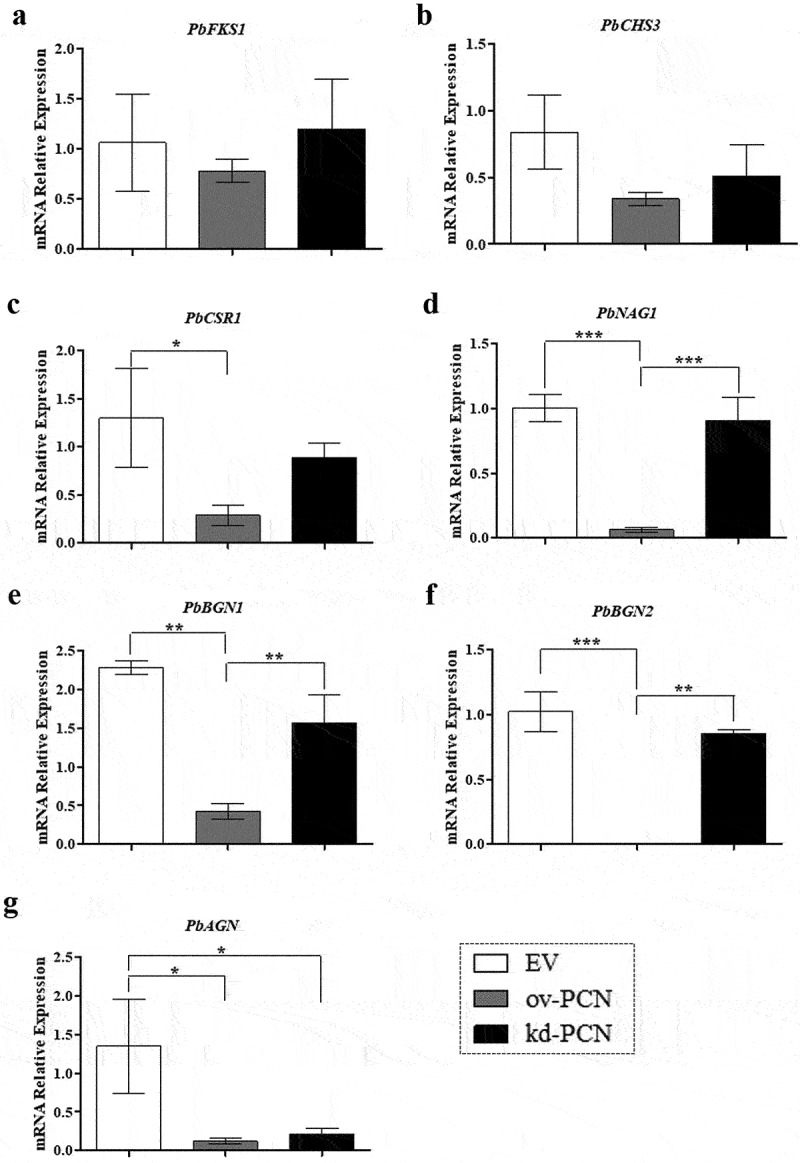
Relative expression of genes related to cell wall remodeling in PCN-transformed *P. brasiliensis* yeasts. the yeasts for RNA extraction were obtained after 72 h of culturing. Samples converted to cDNA were analyzed for the relative expression of *PbFKS1* (a), *PbCHS3* (b), *PbCSR1* (c), *PbNAG1* (d), *PbBGN1* (e), *PbBGN2* (f), and *PbAGN* (g) using RT-quantitative PCR. The Ct values of the transcripts were normalized to the relative expression of the endogenous control L34. Results are expressed as mean ± SD, and the relative expression levels were compared between yeast transformants (ov-PCN or kd-PCN) and the EV-control group. *p < 0.05, **p < 0.001, and ***p < 0.0001.

As PCN overexpression aggravates the disease caused by *P. brasiliensis* through a critical effect on fungal cell wall biogenesis, we investigated whether PCN influences the relative expression of specific transcripts of markers associated with cell wall remodeling in PCN transformants. We included genes related to cell wall synthesis *PbFKS1*, *PbCHS3*, and *PbCSR1* as they encode the enzymes 1,3-β-D-glucan synthase, Chs3 (chitin synthase 3), and Csr1 (chitin synthase regulatory protein), respectively. We also evaluated the expression of genes encoding proteins related to cell wall degradation, such as *PbNAG1* (N-acetyl-β-D-glucosaminidase), *PbBGN1, PbBGN2* (β-1,3-glucanase), and *PbAGN* (α- 1,3-glucanase). After 72 h of culturing, we found no substantial changes in the relative expression levels of *PbFKS1* and *PbCHS3* genes (related to cell wall synthesis) between the transforming strains (ov-PCN or kd-PCN) and the control (EV) (Figures 7(a–b)). Nonetheless, ov-PCN yeasts showed diminished relative expression of *PbCSR1* (Figure 7(c)), a gene encoding one of the putative regulatory proteins of chitin synthase (Csr1). Notably, ov-PCN yeasts, compared with EV control yeasts, remarkably reduced the relative expression levels of all genes related to cell wall degradation (*PbNAG1, PbBGN1, PbBGN2*, and *PbAGN*) (Figures 7(d–g)). Compared to kd-PCN, ov-PCN yeasts showed a drastic reduction in the relative expression of *PbNAG1*, *PbBGN1*, and *PbBGN2* genes (Figures 7(d–f)). We normalized the data concerning the relative expression levels of the transcripts with those of the L34 (Figure 7) and A-Tub genes (data not shown); the results were consistent with the data concerning both endogenous controls.

## Discussion

We demonstrated that PCN-overexpressing *P. brasiliensis* yeasts (ov-PCN) were more susceptible to antifungal agents (amphotericin B, itraconazole, and micafungin) than wild-type yeasts (wt-PCN). This assertion reflects the lower MIC values for ov-PCN than for wt-PCN yeasts that underwent antifungal drug action. The quality of the microdilution assay used for MIC determination was controlled by establishing the MIC values for *C. parapsilosis* under amphotericin B and itraconazole action; the obtained values were consistent with the CLSI standardization [13] (data not shown).

The fungistatic activity of echinocandins (micafungin) results from inhibition of β-(1,3)-glucan synthesis [17]. The β-1, 3-glucan content in the wall of *P. brasiliensis* yeasts was greatly lower (3.9 to 10.6%) than that in mycelial forms (20.2 to 31.4%) [18], justifying the minimal effect of echinocandins on yeasts. We verified that ov-PCN *P. brasiliensis* yeasts are more susceptible to micafungin effects because they exhibit a significantly lower MIC (64 μg.ml^−1^) than wt-PCN yeasts (>128 μg.ml^−1^). This observation raises the question of whether β-1,3-glucan content is higher in the cell wall of ov-PCN than in that of wt-PCN yeasts.

Herein, we showed that kd-PCN and wt-PCN yeasts are susceptible to antifungal agents that act on the fungal membrane or cell wall (amphotericin B, itraconazole, and micafungin). The technology for silencing PCN in *P. brasiliensis* utilizes antisense RNA (AsRNA) and genetic transformation via *A. tumefaciens* (*A. tumefaciens*-mediated transformation, ATMT) [7]. It generated clones 40–60% reduced in the levels of PCN relative expression (mRNA) compared to wt-PCN yeasts. These results suggest that although the reduced levels of PCN expression were sufficient to diminish the separation among yeast cells, making them more clustered [7], they could not confer distinct resistance to kd-PCN yeasts when exposed to antifungal drugs.

Through light microscopy, we demonstrated that wt-yeasts and PCN transformants did not alter their morphology when exposed to amphotericin B and itraconazole. Nonetheless, incubation with 64 µg.ml^−1^ and 128 µg.ml^−1^ micafungin affected the growth and morphology of ov-PCN yeasts and prevented their clustering, compared to the cellular organization pattern shown by the other strains. This observation is consistent with the fact that amphotericin B and itraconazole are drugs that act on the fungal cell membrane, whereas micafungin targets the cell wall, inhibits β-1,3-D-glucan synthase, affects the cell wall composition (β-1,3-glucan), and promotes cell lysis. Our data confirmed that ov-PCN yeasts had augmented β-1,3-glucan content in the cell wall compared to *P. brasiliensis* wt-yeast content (3.9 to 10.6%), as previously reported [18]. These results suggest that the inhibitory effect of micafungin on ov-PCN-transformed yeasts is due to increased β-1,3-glucan content in their cell wall.

Our investigation of the effect of stress-inducing agents on the cell wall of *P. brasiliensis* transformants showed that ov-PCN yeasts are more susceptible to the action of calcofluor and Congo red than the wild-type yeasts, as demonstrated by qualitative and quantitative susceptibility analyses. Hypersensitivity of PCN-overexpressing yeasts to calcofluor may indicate an increase in the polysaccharide content of the cell wall in ov-PCN yeasts. Selvaggini et al. [19] demonstrated that augmented fungal hypersensitivity to calcofluor is closely associated with a high chitin content in the cell wall. We suggest that yeasts that overexpressed PCN, probably through increased chitinolytic activity, displayed cell wall weakening, an alteration that activated compensatory mechanisms to increase chitin and β-1,3-glucan content. Augmentation of cell wall polysaccharide accounts for yeast hypersensitivity to calcofluor and Congo red. In contrast, kd-PCN yeasts were more resistant to calcofluor action due to the reduced relative expression of PCN, which affects fungal cell wall organization, biosynthesis, and remodeling. Briefly, the modified polysaccharide content in the cell wall of kd-PCN yeast results in resistance to calcofluor.

In the last decade, *G. mellonella* larvae have become a convenient host model for experimental paracoccidioidomycosis [14,20–24], meeting the ethical and economic requirements for good animal experimentation practices. We then used this larval model to compare the host damage caused by inoculation with wild-type yeasts and PCN-transformed yeasts. A comparison of larval infections showed that ov-PCN is the most virulent yeast. This led to diminished host resistance, as indicated by the decreased survival rate, higher tissue fungal load, and reduced haemocyte density, confirming previous results from mice infected with the same *P. brasiliensis* strains [8]. The wt-PCN yeast was the second most virulent strain. The virulence of kd-PCN yeasts in larvae was minimal, differing vastly from that verified in infections with ov-PCN and wt-PCN yeasts [7]. We previously demonstrated a similar virulence pattern for the same fungal strains in mice [7,8]. In this case, the murine host minimized the virulence of the kd-PCN yeasts. At the same time, larvae resistance increased, as revealed by the detection of the highest larval survival rate, reduced fungal load in larval tissue, and increased larval density of haemocytes. We also used the larval infection model to investigate the interference of amphotericin B treatment on infection with wt-PCN, ov-PCN, and kd-PCN yeasts. The survival of infected larvae treated or untreated with amphotericin B was studied. Maximal sensitivity to amphotericin B treatment was given by ov-PCN yeasts because infection with them resulted in the highest larval survival (75%). In contrast, infection with wt-PCN and EV yeasts treated with amphotericin B resulted in 50% survival. The kd-PCN yeasts displayed the lowest susceptibility to amphotericin B treatment, as we verified a 25% larval survival rate.

The behavior displayed by PCN-transformed yeasts when infecting mice and *G. mellonella* is probably associated with PCN activities, such as: (i) it affects the separation among yeast cells, and (ii) influences the size of the chitin fragments released during the host-fungus interaction. Fernandes et al. [7] demonstrated that PCN silencing does not affect yeast cell viability and growth and even reduces the separation between yeast cells. Interestingly, kd-PCN yeasts were more clustered than wt-PCN yeasts. It is likely that agglomeration provides protection to yeasts, favoring resistance to antimicrobial agents. Conversely, ov-PCN yeasts exhibit low adhesion among yeast cells, which is attributed to augmented PCN chitinase activity, which promotes intense chitin degradation and decreases the thickness of the fungal cell wall, culminating in fragility and augmented susceptibility to antifungal drugs. Gonçales et al. [8] emphasized that the overexpression of PCN, owing to its N-acetylglucosaminidase activity, promotes chitin hydrolysis to tiny fragments, responsible for increasing macrophage production of IL-10 and M2-type cell polarization, which are characteristically found in severe and disseminated forms of paracoccidioidomycosis. This hypothesis may explain how ov-PCN yeasts lead to *in vivo* exuberant pathogenicity.

We also hypothesized that the exacerbated virulence of ov-PCN yeasts could potentiate the cytotoxic effects of amphotericin B, which could reduce larval haemocyte density. Infection with ov-PCN yeasts, followed by treatment with amphotericin B, maintained 75% larval survival over a 12-day observation period. The data reported by De Lacorte Singulani et al. [14] are similar to ours when correlating the survival curve *vs*. haemocyte density in larvae. Larvae infected with Pb18 yeasts (equal to wt-PCN, 5 × 10^6^ cells/larva) and treated with amphotericin B (2 mg/kg) displayed an 88% survival rate. Despite this, amphotericin B treatment (2 mg/kg) 48 h after the larval infection did not induce recruitment of haemocytes, for which the density was similar to that observed in the control group larvae, which received PBS instead of the antifungal drug.

We investigated the effect of PCN on the anatomopathological features of *G. mellonella* larvae infected with *P. brasiliensis*. We verified that PCN affects the virulence of the yeasts because larval infection with ov-PCN yeasts resulted in a high number of fungal aggregates and disseminated tissue lesions. In contrast, larval infection with kd-PCN yeast resulted in fewer yeast aggregates and less extensive tissue injuries. Notably, PCN overexpression further affected the susceptibility of *P. brasiliensis* yeasts to antifungal treatment, as demonstrated by fewer granuloma-like structures spread in the tissues of larvae infected with ov-PCN yeasts and treated with amphotericin B. The result of this finding is a high frequency of tissue yeast aggregates and extended tissue damage in larvae infected with kd-PCN yeasts and treated with amphotericin B. Studies performed in the *G. mellonella* model of paracoccidioidomycosis indicate that the degree of PCN expression in *P. brasiliensis* yeasts affects *in vivo* fungal virulence and its susceptibility to the antifungal drug amphotericin B.

At the transcriptional level, PCN affects the expression of *P. brasiliensis* genes related to cell wall remodeling, as demonstrated by the reduced *PbCSR1* relative expression in cultured ov-PCN yeasts. *PbCSR1* encodes Csr1, which is one of the three reported putative fungal chitin synthase regulatory proteins (Csr1, Csr2, and Csr3). They are relevant but not essential for yeast viability [25]; Csr1 is important because of its ability to interact with Chs3. This is performed through a prenylation site (CaaX motif), revealed by Csr1 sequence analysis, which indicates its ability to establish protein-protein interactions, probably with Chs3. Grabińska et al. reported that this interaction is significant for Chs3 activity in *Saccharomyces cerevisiae* [26]. We confirmed the presence of a putative chitin synthase regulator (Csr1) in *P. brasiliensis* yeasts, which motivated us to propose that it plays a role similar to that exerted by its analog in *S. cerevisiae* [25], which is essential for Chs3 activity. We have already mentioned that the overexpression of PCN, a protein with chitinolytic activity, results in low thickness and fragility of the cell wall of *P. brasiliensis* yeasts. This modification, in turn, activates compensatory mechanisms capable of increasing chitin content, as evidenced by the hypersensitivity of ov-PCN yeasts to calcofluor action. Transcriptionally, we found that PCN overexpression did not interfere with the relative expression of *PbCHS3* and that the transformant had a low relative expression of *PbCSR1*. Our data are consistent with previous observations in other fungal species, which showed that the transcription levels of genes related to cell wall remodeling do not always correlate with yeast chitin content [27]. The lack of a direct relationship between the measured transcript level and the recovered enzymatic activity occurs because regulation of chitin synthase activity occurs at both the transcriptional and post-transcriptional levels [28]. Niño-Vega et al. [27] reported that the mRNA levels of genes encoding several *P. brasiliensis* chitin synthases (*PbrCHS1, PbrCHS2, PbrCHS4*, and *PbrCHS5*) are higher in hyphae than in yeast. However, yeast cells contain more chitin than mycelial cells. Thus, our data regarding similar levels of *PbCHS3* expression in ov-PCN and wt-yeast may be explained by genome analysis. It revealed seven chitin synthases in *Paracoccidioides* [29], whereas we evaluated only the levels of *PbCHS3* relative expression. Notably, ov-PCN yeasts adopted mechanisms to compensate for the “fragility” of the cell wall, as manifested by the reduced relative expression of genes associated with cell wall degradation (*PbNAG1, PbBGN1, PbBGN2*, and *PbAGN*). The hydrolytic enzymes β-1,3-glucanases and chitinases deserve emphasis because they participate in morphogenetic events to fragment the cell wall structure. Beyond chitinases, N-acetyl-β-D-glucosaminidase should also be highlighted because it is a glycosyl hydrolase that promotes efficient chitin degradation [30]. We showed that PCN overexpression results in low production of N-acetyl-β-D-glucosaminidase, β-1,3-glucanases, and α-1,3-glucanase. Our data reinforce the hypothesis that poor expression of a gene related to cell wall degradation in ov-PCN yeasts may provide a mechanism at the transcriptional level to compensate for the weakening of the cell wall, promoting reduced degradation of polysaccharides that make up the yeast cell wall, that is, chitin, β-1,3-glucan, and α-1,3-glucan. In view of this, further studies are focused on detailed analyzes of the effects of antifungal drugs on the cell wall composition.

The current work demonstrates that PCN overexpression increases the virulence of *P. brasiliensis* yeasts, decreases the *in vitro* and *in vivo* resistance of yeasts to antifungal therapy, and influences cell wall remodeling by reducing the relative expression of genes involved in cell wall degradation. PCN silencing minimized the virulence of yeasts and augmented fungal resistance to the interaction with cells of an alternative invertebrate host, as established by infecting *G. mellonella* larvae with transformed yeasts relative to the endogenous expression of PCN. In conclusion, these findings confirm that endogenous PCN has effects on the virulence and susceptibility to antifungal drugs of *P. brasiliensis* yeasts, and impact on the fungal biology, and the relationship of the yeasts-host cells.

## Supplementary Material

Supplemental Material

## Data Availability

The authors confirm that the data supporting the findings of this study are available within the article.

## References

[1] Ganiko L, Puccia R, Mariano VS, et al. Paracoccin, an N-acetyl-glucosamine-binding lectin of *Paracoccidioides brasiliensis*, is involved in fungal growth. Microbes Infect. 2007;9(6):695–17. DOI:10.1016/j.micinf.2007.02.012 17400504

[2] dos Reis Almeida FB, de Oliveira LL, Valle de Sousa M, et al. Paracoccidioides brasiliensis; purification through affinity with chitin and identification of N-acetyl-beta-D-glucosaminidase activity. Yeast. 2010;27(2):67–76. DOI:10.1002/yea.1731 19908201 PMC3139792

[3] Alegre AC, Oliveira AF, Dos Reis Almeida FB, et al. Recombinant paracoccin reproduces the biological properties of the native protein and induces protective Th1 immunity against *Paracoccidioides brasiliensis* infection. PLoS Negl Trop Dis. 2014;8(4):e2788. DOI:10.1371/journal.pntd.0002788 24743161 PMC3990478

[4] Alegre-Maller AC, Mendonça FC, da Silva TA, et al. Therapeutic administration of recombinant Paracoccin confers protection against *Paracoccidioides brasiliensis* infection: involvement of TLRs. PLoS Negl Trop Dis. 2014;8(12):e3317. DOI:10.1371/journal.pntd.0003317 25474158 PMC4256291

[5] Coltri KC, Casabona-Fortunato AS, Gennari-Cardoso ML, et al. Paracoccin, a GlcNac-binding lectin from *Paracoccidioides brasiliensis*, binds to laminin and induces TNF-α production by macrophages. Microbes Infect. 2006;8(3):704–713. DOI:10.1016/j.micinf.2005.09.008 16476564

[6] Freitas MS, Oliveira AF, da Silva TA, et al. Paracoccin induces M1 polarization of macrophages via interaction with TLR4. Front Microbiol. 2016;7:1003. 27458431 10.3389/fmicb.2016.01003 PMC4932198

[7] Fernandes FF, Oliveira AF, Landgraf TN, et al. Impact of paracoccin gene silencing on *Paracoccidioides brasiliensis* virulence. MBio. 2017;8(4):e00537–17. 28720727 10.1128/mBio.00537-17 PMC5516250

[8] Gonçales RA, Ricci-Azevedo R, Vieira VCS, et al. Paracoccin overexpression in *Paracoccidioides brasiliensis* enhances fungal virulence by remodeling chitin properties of the cell wall. J Infect Dis. 2021;224(1):164–174. DOI:10.1093/infdis/jiaa707 33201217

[9] Maleki A, Roohvand F, Tajerzadeh H, et al. High expression of methylotrophic yeast-derived recombinant human erythropoietin in a pH-controlled batch system. Avicenna J Med Biotechnol. 2010;2(4):197–206. 23407145 PMC3558167

[10] de Paula e Silva AC, Oliveira HC, Silva JF, et al. Microplate Alamar blue assay for *Paracoccidioides* susceptibility testing. J Clin Microbiol. 2013;51(4):1250–1252. DOI:10.1128/JCM.02914-12 23345296 PMC3666786

[11] Palanco AC, Lacorte Singulani J, Costa-Orlandi CB, et al. Activity of 3′-hydroxychalcone against *Cryptococcus gattii* and toxicity, and efficacy in alternative animal models. Future Microbiol. 2017;12(13):1123–1134. DOI:10.2217/fmb-2017-0062 28876122

[12] Tomazett PK, Castro Nda S, Lenzi HL, et al. Response of *Paracoccidioides brasiliensis* Pb01 to stressor agents and cell wall osmoregulators. Fungal Biol. 2011;115(1):62–69. DOI:10.1016/j.funbio.2010.10.005 21215956

[13] CLSI, Clinical and Laboratory Standards Institute. Reference Method for Broth Dilution Antifungal Susceptibility Testing of Yeasts: 4th Informational Supplement-CLSI Document M27-S4-Wayne, PA; 2012.

[14] de Lacorte Singulani J, Scorzoni L, de Paula E, et al. Evaluation of the efficacy of antifungal drugs against *Paracoccidioides brasiliensis* and *Paracoccidioides lutzii* in a *Galleria mellonella* model. Int J Antimicrob Agents. 2016;48(3):292–297. DOI:10.1016/j.ijantimicag.2016.05.012 27444116

[15] Pitangui NS, Fernandes FF, Gonçales RA, et al. Paracoccin: purification and validation of its lectin and enzymatic properties. Methods Mol Biol. 2020;2132:139–149. 32306322 10.1007/978-1-0716-0430-4_14

[16] Almeida F, Antoniêto AC, Pessoni AM, et al. Influence of N-glycans on expression of cell wall remodeling related genes in *Paracoccidioides brasiliensis* yeast cells. 2016;17(2):112–118. DOI:10.2174/1389202917666151116212705 PMC4864839 27226767

[17] Walker LA, Gow NA, Munro CA. Fungal echinocandin resistance. Fungal Genet Biol. 2010;47(2):117–126. 19770064 10.1016/j.fgb.2009.09.003 PMC2812698

[18] Rodríguez-Brito S, Niño-Vega G, San-Blas G. Caspofungin affects growth of *Paracoccidioides brasiliensis* in both morphological phases. Antimicrob Agents Chemother. 2010;54(12):5391–5394. 20937789 10.1128/AAC.00617-10 PMC2981295

[19] Selvaggini S, Munro CA, Paschoud S, et al. Independent regulation of chitin synthase and chitinase activity in *Candida albicans* and *Saccharomyces cerevisiae*. Microbiol. 2004;150(4):921–928. PMID: 15073301:10.1099/mic.0.26661-0 15073301

[20] Thomaz L, García-Rodas R, Guimarães AJ, et al. *Galleria mellonella* as a model host to study *Paracoccidioides lutzii* and *Histoplasma capsulatum*. Virulence. 2013;4(2):139–146. DOI:10.4161/viru.23047 23302787 PMC3654612

[21] Scorzoni L, de Paula e Silva AC, Singulani J de L, et al. Comparison of virulence between *Paracoccidioides brasiliensis* and *Paracoccidioides lutzii* using *Galleria mellonella* as a host model. Virulence. 2015;6(8):766–776. DOI:10.1080/21505594.2015.1085277 26552324 PMC4826127

[22] de Oliveira HC, da Silva J de F, Scorzoni L, et al. Importance of adhesins in virulence of *Paracoccidioides* spp. Front Microbiol. 2015;6:303. 25914695 10.3389/fmicb.2015.00303 PMC4392702

[23] Marcos CM, Tamer G, de Oliveira HC, et al. Down-regulation of TUFM impairs host cell interaction and virulence by *Paracoccidioides brasiliensis*. Sci Rep. 2019;9(1):17206. DOI:10.1038/s41598-019-51540-y 31748561 PMC6868139

[24] Scorzoni L, Alves de Paula e Silva AC, de Oliveira HC, et al. *In Vitro* and *In Vivo* effect of peptides derived from 14-3-3 *Paracoccidioides* spp protein. J Fungi (Basel). 2021;7(1):52. DOI:10.3390/jof7010052 33451062 PMC7828505

[25] Barreto L, Sorais F, Salazar V, et al. Expression of *Paracoccidioides brasiliensis* CHS3 in a *Saccharomyces cerevisiae* chs3 null mutant demonstrates its functionality as a chitin synthase gene. Yeast. 2010;27(5):293–300. DOI:10.1002/yea.1748 20037924

[26] Grabińska KA, Magnelli P, Robbins PW. Prenylation of *Saccharomyces cerevisiae* Chs4p affects Chitin Synthase III activity and chitin chain length. Eukaryot Cell. 2007;6(2):328–336. 17142567 10.1128/EC.00203-06 PMC1797950

[27] Niño-Vega GA, Munro CA, San-Blas G, et al. Differential expression of chitin synthase genes during temperature-induced dimorphic transitions in *Paracoccidioides brasiliensis*. Med Mycol. 2000;38(1):31–39. DOI:10.1080/mmy.38.1.31.39 10746225

[28] Choi WJ, Santos B, Durán A, et al. Are yeast chitin synthases regulated at the transcriptional or the posttranslational level? Mol Cell Biol. 1994;14(12):7685–7694. DOI:10.1128/mcb.14.12.7685-7694 7969112 PMC359310

[29] Puccia R, Vallejo MC, Matsuo AL, et al. The *Paracoccidioides* cell wall: past and present layers toward understanding interaction with the host. Front Microbiol. 2011;20:257. 10.3389/fmicb.2011.00257 PMC3243086 22194733

[30] Tomazett PK, Cruz AH, Bonfim SM, et al. The cell wall of *Paracoccidioides brasiliensis*: insights from its transcriptome. Genet Mol Res. 2005;4(2):309–325. 16110448

