# Supplementary material for: Role of paracoccin on Paracoccidioides brasiliensis virulence and susceptibility to antifungal drugs in the Galleria mellonella larvae model
Source: Virulence. 2023 Jan 4;14(1):2150455. doi: 10.1080/21505594.2022.2150455 (PMC9815234; doi:10.1080/21505594.2022.2150455)
Supplement: Supplemental Material [file KVIR_A_2150455_SM6115.docx]

**SUPPLEMENTARY MATERIAL**


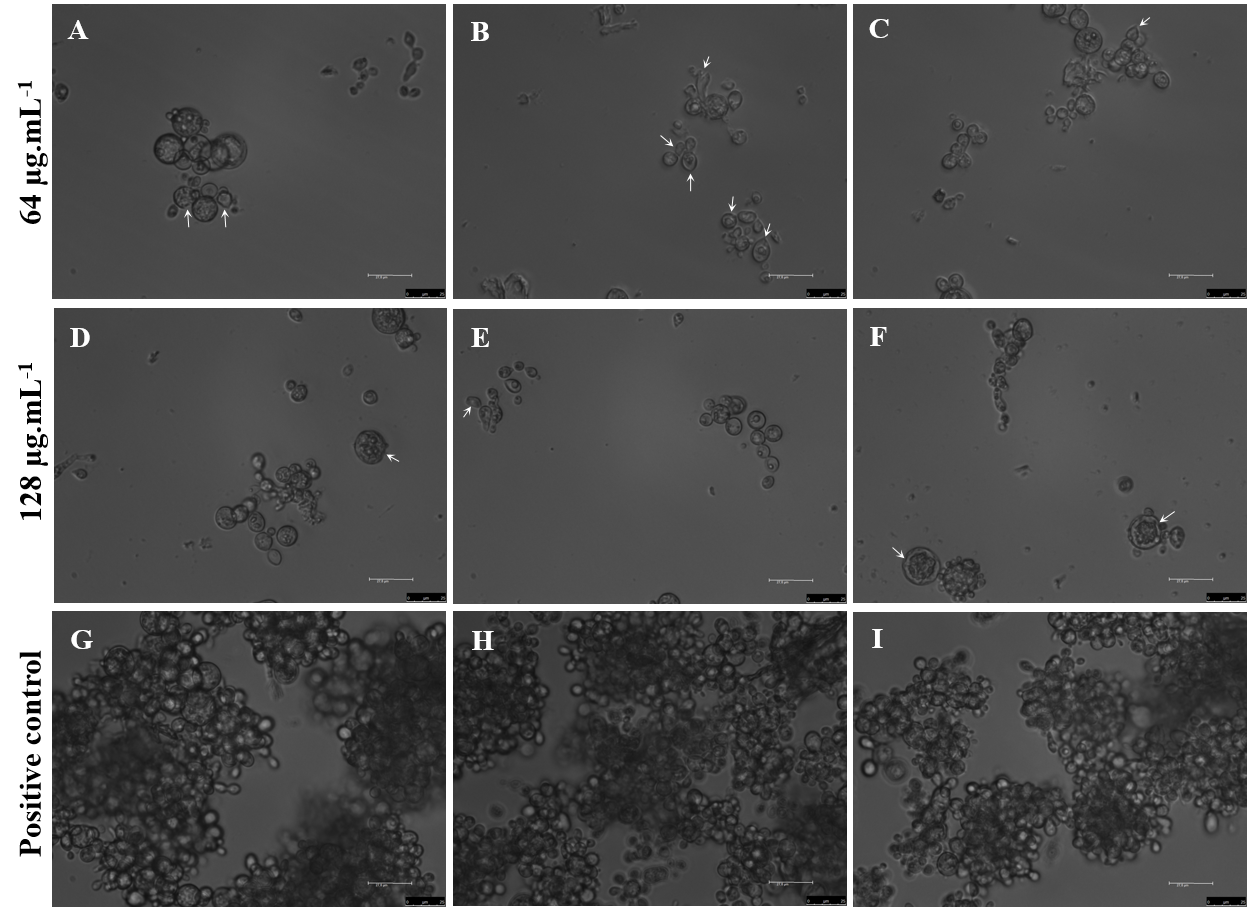


**Figure S1. ov-PCN showed disordered cell structure and altered general pattern of yeast organization by the action of micafungin.** PCN-overregulated strain (ov-PCN) were incubated with 64 µg.mL^-1^ (**A**, **B** and **C**) and 128 µg.mL^-1^ (**D**, **E** and **F**) micafungin for 72 h. Positive control: growth control, yeast free from drug effects (**G**, **H** and **I**). 63X objective and bars, 50 µm. White arrows indicate the damage to integrity of cell membrane.
